# Influence of Alkali Cations on Redox Matching and Capacity Access in Redox-Mediated Flow Batteries

**DOI:** 10.1021/acsomega.5c08835

**Published:** 2025-12-31

**Authors:** Eylul Ergun, Daniel Rourke, Shabdiki Chaurasia, Tulsi M. Poudel, Patrick J. Cappillino, Ertan Agar

**Affiliations:** † Department of Mechanical Engineering, Energy Engineering Graduate Program, 14710 University of Massachusetts Lowell, Lowell, Massachusetts 01854, USA; ‡ Department of Chemistry and Biochemistry, 14709 University of Massachusetts Dartmouth, Dartmouth, Massachusetts 02747, USA

## Abstract

Cost-effective redox flow batteries (RFBs) offer reliable energy storage for intermittent solar and wind sources; however, their energy density is inherently lower than that of lithium-ion batteries because of solubility limitations. The redox-mediated flow battery (RMFB) concept addresses this limitation by combining the operational flexibility of RFBs with the high energy density of solid-state batteries. In this system, a solid material which is immobilized inside the electrolyte (the booster) undergoes charge/discharge indirectly through electron transfer mediated by a dissolved active species (mediator). As a result, the energy density of the RMFB is ideally determined by the amount of solid material incorporated. Beyond booster engineering and material screening, the intercalating cation to the booster upon discharge is able to aid in this potential alignment. In this work, electrochemical techniques including cyclic voltammetry (CV), electrochemical impedance spectroscopy (EIS), galvanostatic charge–discharge (GCD), and operando ultramicroelectrode cyclic voltammetry (UME-CV) are employed to identify the optimal utilization window of the booster and quantify trends in redox mediation kinetics by comparing the impact of three alkali cations on redox mediation in Prussian blue (booster)/ferri/ferrocyanide (mediator) systems. The results indicate that Li^+^ and Na^+^ diffuse more slowly within the booster compared to K^+^, leading to superior intercalation dynamics with K^+^. Under the tested conditions with varying mediator concentrations and cation species, the maximum booster utilization was found to be 35% using 200 mM potassium ferri/ferrocyanide. These findings highlight that achieving higher energy densities in RMFBs requires careful optimization of mediator concentration, electrolyte composition, and redox mediation kinetics.

## Graphical abstract

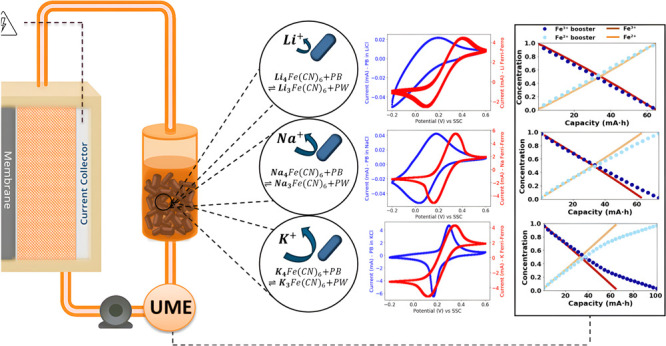

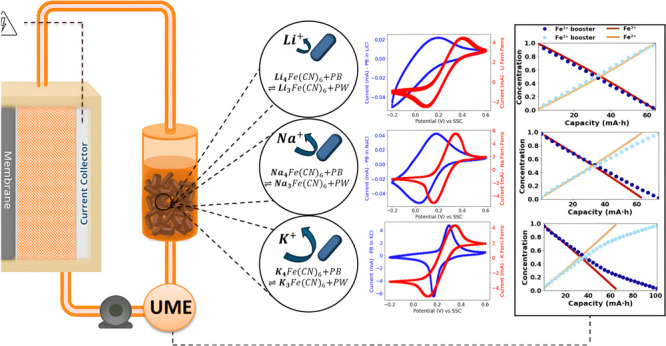

## Introduction

Global warming is one of the most pressing challenges of this century, driven primarily by greenhouse gas emissions. CO_2_ makes up a large proportion of these emissions, most of which originate from fossil fuels that currently generate about two-thirds of the world’s electricity.^1–3^ To limit environmental harm and protect the ecosystem, the energy sector is transitioning toward renewable energy sources including solar and wind.^4,5^ These systems offer reliable electricity generation and are central to efforts to decarbonize power grids.^6,7^ However, solar and wind are intermittent power sources, which require energy storage solutions to mitigate fluctuations in their power output and ensure a reliable energy supply.^8^ Redox flow batteries (RFB) provide cost-effective, reliable long-term energy storage decoupling energy and power, making them ideal for large-scale sustainable applications with reduced carbon footprint. However, RFB energy density is inherently limited by the solubility of the active species, making them less energy dense compared to their current competitor: conventional lithium ion batteries.^9,10^ Achieving the widespread implementation of RFBs while realizing their cost advantages requires increasing their capacity delivered per unit of dissolved active material used.^11^ To address this critical bottleneck, an innovative strategy is to immobilize energy-dense solid materials (booster) inside the external tanks, thereby leveraging the high energy density of solid state batteries.

### Redox-Mediated Flow Batteries

Achieving high energy densities is possible for conventional RFBs by increasing the solubility of the active species undergoing oxidation and reduction in the flow cell. However, high concentrations of active species often result in increased viscosities restricting fluid flow and ion mobility. Because transport properties (e.g., viscosity, diffusion coefficient, conductivity) are not independent, changes in one property will inevitably affect the other.^12^ As a result, RFB operation requires carefully optimizing the active species concentration: excessive viscosity lowers ion mobility, leading to greater mass and charge transfer losses, while also increasing the pumping energy required to circulate the electrolyte.^12–14^ The redox-mediated flow battery (RMFB) concept, often referred to as redox targeting flow battery, addresses the solubility limitations by enabling energy storage in solid materials stored in external tanks, decoupling capacity from dissolved species concentration. This process follows a two-step electrochemical mechanism. In the first step, the dissolved active species (mediator) undergoes electrochemical reactions at the electrodes. It is then circulated to the external tanks, where the booster is located, and transfers electrons to or from the booster, driven by the potential difference between them. This electron exchange charges or discharges the booster accordingly.^15^

The first RMFB concepts used two mediators: one with a higher redox potential than the booster for charging, and another with a lower potential for discharging.^16^ Later, LiFePO_4_ (LFP) was shown to undergo both lithiation and delithiation with ferrocene-based mediators,^17^ while TiO_2_ charge/discharging was achieved using bis­(pentamethylcyclopentadienyl)­cobalt (CoCp*_2_) and cobaltocene (CoCp_2_) as mediators.^18^ These advances led to the first redox-mediated lithium battery, delivering high energy densities with relatively low mediator concentrations.^15,19^ Subsequently it was noted that, although dual mediators can exploit booster capacity, overpotentials can lead to voltage losses.^20^ Studies have shown single mediators like iodine can enable both processes, as LFP’s potential lies between the iodine couples.^21^ Beyond lithium, polyaniline in a vanadium/iron flow battery used Fe^2+^/Fe^3+^ as mediator.^22^ Following the first full-cell demonstrations, significant modeling efforts have further advanced the RMFB field by linking solid–mediator kinetics, reactor design, and cell performance.^20,23–26^ Over the years, the concept has been extensively validated through numerous experimental studies focusing on lithium-ion battery cathode materials, functional polymers, and Prussian blue analogues (PBA), largely confirming their potential as effective boosters for large-scale applications.^27–44^

### Redox Potential Matching and State of Charge (SoC) Window

To enhance booster utilization without incurring overpotential losses, it is advantageous to identify a single Nernstian mediator whose redox potential closely matches that of the solid booster.^20,22,24,27,29,45^ While introducing a booster to the external tank removes the capacity limitation imposed by the solubility of the mediator, the operating potential range of the flow battery is still determined by the formal potential of the dissolved mediator in the electrochemical cell. This means the highest and lowest potential that the battery can reach is set by the redox potential window of the mediator, not the booster. Accordingly, the SoC profile of the booster may extend beyond the mediator’s redox potential limits, meaning that part of the booster’s capacity cannot be accessed because portions of its redox profile fall outside the electrochemically accessible window during normal operation. Mismatch between the mediator’s redox window and the booster’s potential range reduces the fraction of the booster’s theoretical capacity that can be utilized in practice since charge transfer is restricted to only the overlapping region.^24^

Therefore, in a system where both the booster and mediator exhibit Nernstian potential profiles, a close match between their redox potentials is essential in single-mediator systems. Under these conditions, any deviation in activity of the materials will drive charge transfer between the booster and mediator without significant overpotentials, enabling improved capacity utilization of the booster.^26,27,46^

### Strategies for Matching Redox Potentials

Achieving this match typically begins by screening commonly used flow battery mediators and well-known solid-state battery materials as boosters.^16–19,21,22,27–30,33,34,36,38,42–44,47,48^ Polymer-based boosters can be fabricated from common RFB active materials,^35,49^ while inorganic boosters with metal redox centers can be tuned via doping or redox-center modification. PBAs, canonically composed of Fe^2+^/Fe^3+^ centers, can be partially or fully substituted with other transition metals (Co, Mn, Ni, Cu), shifting its redox potentials.^50,51^ With a suitable mediator, the PBA’s potential can thus be tuned for optimal performance. Similarly, for LFP-based boosters, Cai et al. demonstrated that manganese doping effectively tunes the composition of the booster, allowing controlled adjustments of its redox potential.^39^

Our earlier work provided direct experimental evidence of how compositional tuning, even through unintentional ion introduction, can influence performance. We observed substantial capacity gains when pairing a mushroom-inspired vanadium mediator with cobalt hexacyanoferrate (CoHCF). Surprisingly, cyclic voltammetry (CV) revealed a potential mismatch between the mediator and CoHCF, despite literature indicating well-aligned potentials. Further analysis traced the source of this shift to trace potassium ions introduced during CoHCF synthesis.^42^ This observation is consistent with established cation-size effects on redox intercalation potentials, demonstrating that even minor cation impurities can shift redox potentials and impact capacity.^52^

In existing literature, research predominantly emphasizes the interactions and reactions between different active materials in redox-mediated systems, rather than systematically investigating the isolated effects of individual electrolyte parameters. Notably, only a limited number of studies have addressed this gap.^41,48^ For example, the low utilization of polymer bead boosters has been attributed to the ionic strength of the electrolyte. Through a carefully controlled, systematic study, Asserghine and colleagues revealed that the electrolyte uptake of polymer-based boosters is significantly affected by the concentration of the supporting salt. They demonstrated that decreasing salt concentration can enhance booster utilization to as much as 92%.^41^ These findings stress that the efficiency of redox-mediation is not determined solely by mediator–booster compatibility or intrinsic material properties but is also critically dependent on broader electrolyte conditions. Such investigations are crucial for targeted optimization of RMFBs, offering practical guidelines for designing electrolyte formulations that maximize performance, durability, and scalability. The absence of this kind of systematic work represents a significant barrier to advancing the field from proof-of-concept demonstrations toward optimized, application-oriented RMFB systems.

In addition to electrolyte composition, another underexplored but potentially powerful lever for tuning RMFB performance lies in tailoring the identity of the intercalating cation itself. The literature documents that intercalation-based solid battery materials can exhibit shifts in redox potential that depend on the ionic size of the intercalating cation.^52,53^ Therefore, beyond the commonly explored chemical tuning of redox mediators and solid boosters, tailoring the intercalation cation itself presents an alternative and promising strategy to modulate the electrochemical properties of the system. By selecting different cations for intercalation, it is possible to fine-tune the redox potential and improve compatibility between the mediator and booster, which could enhance overall reaction efficiency and battery performance.

Motivated by these considerations and inspired by the findings from our previous work, we undertook a systematic study to investigate the effects of intercalating cations in intercalation-based boosters. Recognizing that RMFBs engage in a complex interplay of electrochemical and chemical steps where not only the redox couples, but also the redox-inactive ions in the electrolyte, can significantly influence kinetics and thermodynamics, we selected a widely used booster/mediator pair, ferri/ferrocyanide as the mediator and Prussian Blue (PB) as the booster. By systematically investigating the influence of cations of different sizes on redox-mediated reactions in aqueous RMFBs, we aim to understand the ideal thermodynamic limits for utilization, practical utilizations for boosters and redox mediation rate upon charging and discharging. In a separate study, currently in submission alongside this work, we report on a nonaqueous RMFB system in which we directly probe the heterogeneous mediator/booster redox reactions.^54^

## Results and Discussion

Three alkali metal cations; lithium (Li^+^), sodium (Na^+^), and potassium (K^+^) were investigated in an aqueous environment to understand their effects on RMFB performance. Ferri/ferrocyanide was chosen as the mediator due to its well-established electrochemical stability and rapid redox kinetics, making it an ideal reference system.^55,56^ PB was selected as the booster because it is widely used in conjunction with ferri/ferrocyanide in flow battery systems, offering complementary redox behavior.^25,28,38,48^ Additionally, the alkali metal analogues of ferri/ferrocyanide mediators are either commercially available or can be readily synthesized, facilitating systematic comparison across different cation environments.

### Cation-Dependent Redox Potentials

Cyclic voltammetry (CV) experiments presented in Figure 1 provide a clear view of how closely the redox potentials of the mediator and the booster align which is a key factor for efficient redox mediation. As shown in Figure 1a, potassium ferri/ferrocyanide, a commonly used mediator, exhibits redox potentials that are well matched with those of PB.

**Figure 1:**
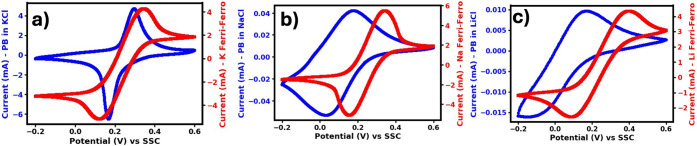
Cyclic voltammetry profiles of 0.1 M ferri/ferrocyanide (red) and Prussian blue in 1 M supporting salt (blue), the potentials are recorded against Ag/AgCl (SSC): a) potassium ferri/ferrocyanide and Prussian blue in KCl, b) sodium ferri/ferrocyanide and Prussian blue in NaCl, and c) lithium ferri/ferrocyanide and Prussian blue in LiCl.

Sodium ferri/ferrocyanide indicated with the red CV profile in Figure 1b displays a slightly more positive redox potential than its potassium analogue, while the lithium variant (Figure 1c) exhibits a potential very similar to that of the sodium form. Overall, the mediator’s redox potential remains relatively consistent across different cation environments, as indicated by the red CV profiles.

In sharp contrast to the mediator, the redox potential of PB changes markedly depending on the intercalating cation, displaying a clear negative shift that systematically follows the trend of increasing alkali ion size.^57^ This means that the booster’s electrochemical behavior is not fixed, but rather is directly governed by which cation occupies its lattice.^58^ Importantly, perfect matching between booster and mediator redox potentials is observed only when potassium (K^+^) is the intercalating ion, conditions under which charge transfer can proceed with minimal overpotential and high efficiency.

When sodium (Na^+^) is present, PB’s redox potential shifts to more negative values relative to the mediator, thereby weakening this thermodynamic match. The effect becomes even more pronounced with lithium (Li^+^), which causes the greatest negative shift of all, producing the largest mismatch and potentially the highest associated losses.

Figure 2 presents the thermodynamic limitations and utilization window of the booster, plotted alongside the voltage window of the ferri/ferrocyanide analogues. To characterize the booster’s performance in different cation environments, a three-electrode setup with a booster-coated glassy carbon electrode in a solution of 1 M corresponding chloride salt was used to obtain its charge/discharge profile. The resulting booster SoC profiles were then superimposed on the mediator’s SoC curve, which was calculated using the Nernst equation. To replicate practical conditions and observe any intrinsic behavior, the booster was charged and discharged under a slow galvanostatic protocol using a constant current of ±0.05 mA. Even under such conditions, the voltage hysteresis did not vanish, indicating that this behavior is highly likely to persist in practice.^59^ While the specific mechanisms underlying the hysteresis are not discussed here, the key point is that it remains present even at slow scan rates and would likewise be observed in flow-battery operation. Potassium-based electrolyte exhibited a relatively flat voltage profile across a broad SoC range, deviating somewhat from ideal Nernstian behavior but remaining within the battery’s operational potential window.^46^ In contrast, the sodium-based electrolyte showed a much steeper voltage curve, exceeding the potential window of the ferri/ferrocyanide calculated using the Nernst equation. Similarly, the lithium-based electrolyte displayed a steep profile accompanied by increased hysteresis between charge and discharge cycles. For comparison, these results are evaluated against the ferri/ferrocyanide mediator by referencing its well-characterized Nernst potential profile, which serves as a common catholyte benchmark in flow batteries. These profiles in Figure 2 also illustrate the extent to which the booster can be charged/discharged while the mediator is cycled between 0 and 100% state of charge (SoC). Such visualization aids in developing intuition about the charge and discharge pathways, which differs for boosters containing different intercalating cations.

**Figure 2:**
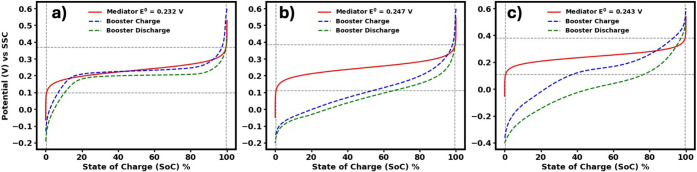
Electrolyte state of charge (SoC) as a function of potential vs SSC (Ag/AgCl in 1 M KCl), calculated using the Nernst equation and experimental half-wave potentials (red), compared with the experimental SoC of Prussian blue during galvanostatic charging (blue) and discharging (green). The gray lines represent the accessible SoC window of the booster. Data are shown for a) potassium ferri/ferrocyanide and Prussian blue in 1 M KCl, b) sodium ferri/ferrocyanide and Prussian blue in 1 M NaCl, and c) lithium ferri/ferrocyanide and Prussian blue in 1 M LiCl.

As indicated by the gray lines in Figure 2, the booster can ideally be cycled only within that defined voltage and SoC range. Figure 3 presents the ideal booster utilization window by plotting the booster’s state of charge (SoC) against that of the mediator, utilizing the charge–discharge profiles from the characterization study shown in Figure 2. When potassium is used as the intercalating cation, the voltage corresponding to 0% mediator SoC aligns with that at approximately 10% booster SoC. This indicates that a fully discharged mediator can only lower the booster potential to a level equivalent to its 10% SoC, rather than fully depleting it. On the other end of the spectrum, the fully charged mediator reaches a potential nearly identical to that of the booster at 100% SoC. Together, these relationships define an operational range for Prussian Blue (PB) between 10% and 100% SoC. In contrast, with sodium and lithium as intercalating cations, the operational range is narrower, with cycling occurring between 60% and 100% SoC theoretically. Although both Na^+^ and Li^+^ environments yield a SoC utilization of 40%, it is important to note that a booster with a lower redox potential is designed primarily to assist the mediator regeneration during discharge as it is established in dual mediator systems. Therefore, solely based on charge/discharge curves, the booster’s effect is expected to be most prominent during discharge in the Na^+^ and Li^+^ environment.

**Figure 3:**
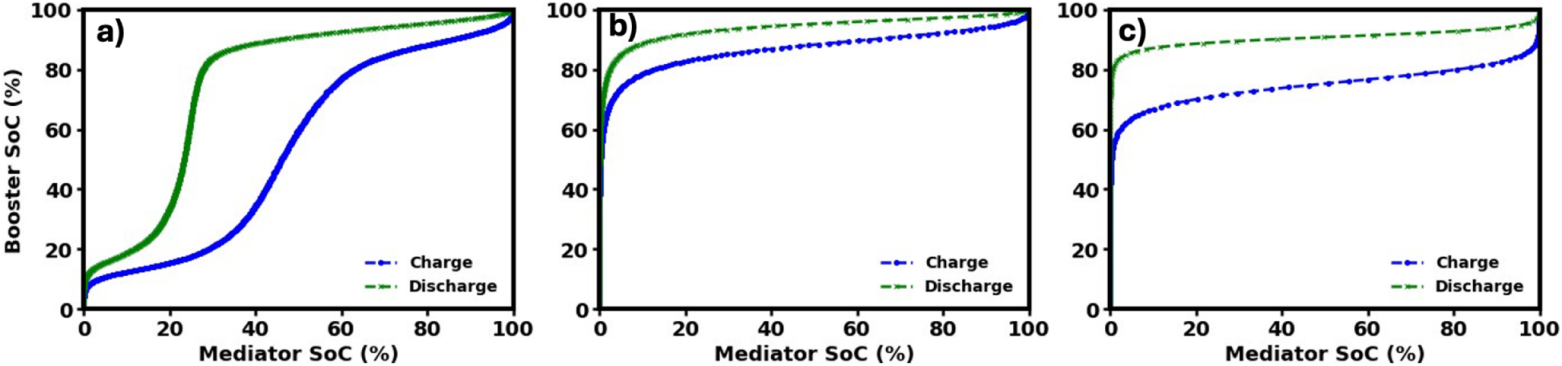
State of charge (SoC) of Prussian blue as a function of the SoC of the mediator for different electrolyte cations: a) K^+^, b) Na^+^, and c) Li^+^.

### Symmetric-Cell Capacity Gains

To move beyond static measurements after establishing the ideal utilization windows for the RMFB system with different cations, symmetric cell cycling experiments were performed to investigate system behavior within a flow battery architecture. These experiments employed a compositionally symmetric, volumetrically asymmetric setup.^60^ By minimizing the volume on one side of the flow battery, that compartment was deliberately constrained to be capacity-limiting, allowing its behavior to be closely monitored while avoiding the situation in which the opposite side would gradually become capacity-limiting during cycling. To assess the impact of introducing boosters, baseline performance was established by cycling the flow battery several times without any booster present in the tanks. Following the baseline cycles for each cation environment and its different concentrations, 2.6 to 3.0 g of booster was added to 25 mL of electrolyte. For a fair comparison of capacity enhancements, focus was placed on the third discharge cycle, ensuring the booster had sufficient time for complete wetting and integration.

The efficiency of redox mediation is a known factor influencing booster utilization, with a faster mediation reaction rate often correlating with higher concentrations.^26,48^ In this study, mediator concentrations varied between 100 mM and 200 mM, which resulted in a maximum booster utilization of only 35%. These findings suggest that higher mediator concentrations are likely necessary to achieve optimal utilization. Moreover, the size of the booster particles and its microstructure may further limit utilization.^61^ Additionally, the rate is significantly influenced by the design of the RMFB, which is inherently complex due to the presence of two competing reactions, one occurring in the external tanks and the other within the flow cell. Because the reaction kinetics inside the tanks cannot always keep pace with those in the flow cell, slower charge and discharge schedules are needed.^23^ A constant current density of 10 mA/cm^2^ was applied during the RMFB charge/discharge. While a slower current could have potentially improved the utilization of the booster, the chosen operational flow battery current density was sufficient to enable a direct and comparative evaluation of the different cations.

Thermodynamic data suggests that K^+^ should enable higher utilization values, whereas Na^+^ and Li^+^ are expected to show comparable to each other but lower utilization, although, as our results indicate, these expectations do not fully capture the behavior of the complete RMFB system. Comparing the trends in utilization across the tested systems, in a potassium-based environment (Figure 4—indicated with blue bars), the utilization of PB increases with rising mediator concentration, which is consistent with observations reported in the literature.^26,48^ Capacity enhancement in sodium environments remained limited (Figure 4—indicated with orange bars), achieving only about 10 mA·h per unit of PB even at the highest mediator concentration tested. Similarly, in lithium-based electrolytes (Figure 4—indicated with green bars), increasing mediator concentration did not result in significant capacity gains. These outcomes suggest that capacity improvements with higher mediator concentrations are strongly dependent on the intercalating ion type. The pronounced benefits observed primarily in potassium systems highlight the importance of ion-specific interactions affecting redox-mediation efficiency. Surprisingly, lithium based electrolyte deviate from ideal thermodynamic expectations and show minimal participation in redox-mediated reactions, indicating possible kinetic or structural barriers that limit mediator-booster interactions in lithium environments.

**Figure 4:**
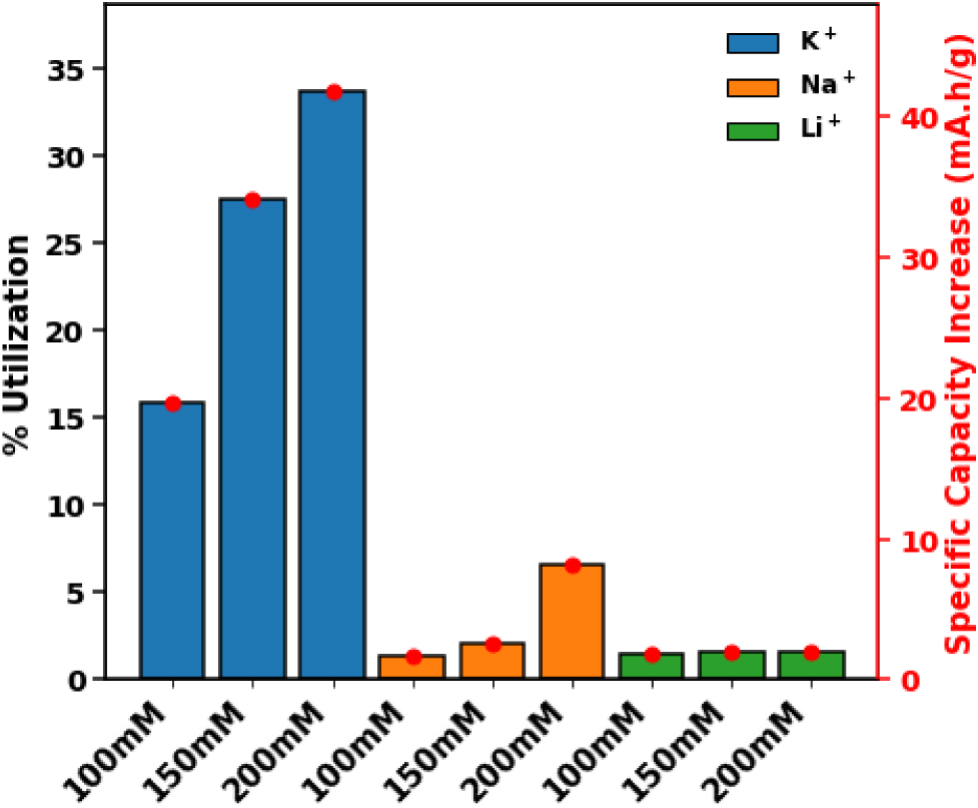
Percent booster utilization across various mediator concentrations and different mediator analogs. Capacity gain per gram of booster is indicated by red markers.

### Operando Ultramicroelectrode Current Partitioning

The reaction rates occurring inside the tank, as well as their evolution over time, cannot be determined solely from flow-cell charge/discharge profiles or booster-utilization data. To deconvolute the contributions from reactions within the tank and those at the electrodes, operando ultramicroelectrodes (UMEs) were employed. By performing CV with UMEs, the mediator SoC was monitored in real time during flow-battery cycling, in a manner consistent with previous reports.^62^ Preliminary experiments of the UME CV method demonstrated that the height difference between the negative and positive steady-state CV plateaus remained constant over several cycles, both with and without the booster. This observed stability indicates consistent mediator concentration, thereby ruling out any significant mediator crossover or side reactions within the system. This validation provides a strong foundation for our central assumption: any observed capacity gain is solely a result of the booster’s contribution. Therefore, while the change in charge for booster is not directly probed, the UME CV data on mediator stability provides strong evidence that the difference between the total current applied to the cell ${\left[\frac{dQ}{dt}\right]}_{\mathrm{total}}$ and the current used by the mediator ${\left[\frac{dQ}{dt}\right]}_{\mathrm{mediator}}$ can be attributed to the booster.

Through this approach, the redox-mediation reaction is observed by quantifying both the current used to oxidize and reduce the mediator and the current transferred to the booster material. The battery capacity was obtained from RMFB symmetric cell cycling at a given *t*_1_ and *t*_2_, the total rate of capacity change (current (mA)) ${\left[\frac{dQ}{dt}\right]}_{\mathrm{total}}$ was then calculated using eq 1

1 $${\left[\frac{dQ}{dt}\right]}_{\mathrm{total}}=\frac{{\left(\mathrm{Capacity}\right)}_{t_{2}}-{\left(\mathrm{Capacity}\right)}_{t_{1}}}{t_{2}-t_{1}}\times 3600$$

The change in mediator concentration, tracked using operando UME CV, was converted into a change in capacity by multiplying it by the Faradays constant (*F*) and volume of the electrolyte (*V*). The current used by the mediator was then calculated from this relationship accordingly.

2 $${\left[\frac{dQ}{dt}\right]}_{\mathrm{mediator}}=\frac{{\left({[\mathrm{Fe}}^{3+}\right]}_{t_{2}}-{{[\mathrm{Fe}}^{3+}]}_{t_{1}})\times F\times V}{t_{2}-t_{1}}\times 3600$$

Any excess current (calculated by subtracting eq 2 from eq 1) represents the current utilized by the booster.

$${\left[\frac{dQ}{dt}\right]}_{\mathrm{booster}}={\left[\frac{dQ}{dt}\right]}_{\mathrm{total}}-{\left[\frac{dQ}{dt}\right]}_{\mathrm{mediator}}$$ 3

The quantification is represented in Figure 5 in terms of $\left[\frac{dQ}{dt}\right]$ as a function of the mediator SoC, during constant current charging (Figure 5a) and discharging (Figure 5b). This evaluation clearly demonstrates distinct behaviors for the sodium, lithium, and potassium analogs of the mediator during RMFB operation.

**Figure 5:**
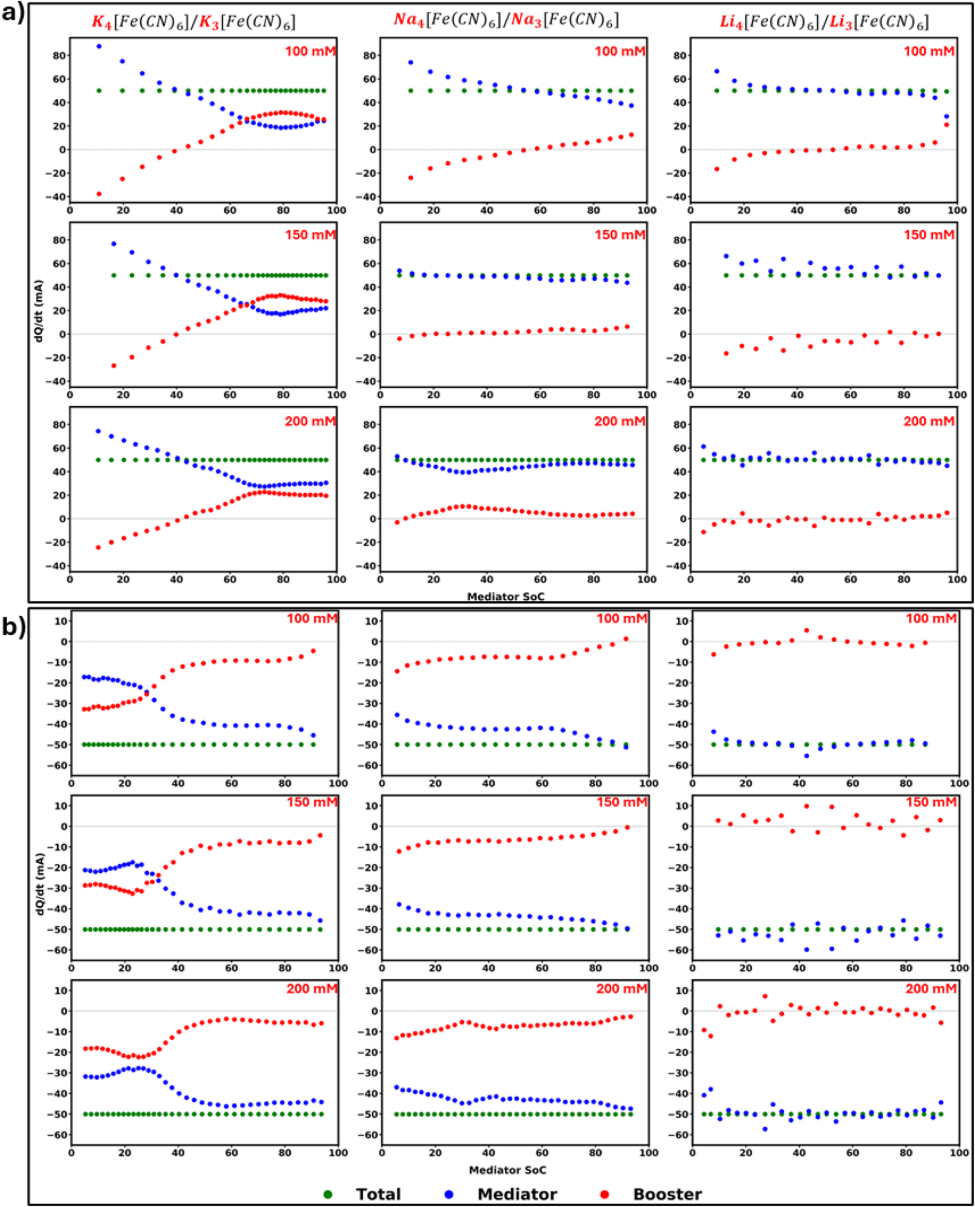
Rate of charge transfer between the mediator and the booster as a function of the mediator’s state of charge. Total applied current to the flow cell is shown with green markers; the fraction consumed by mediator oxidation/reduction is shown in red, and the remainder transferred to the booster is shown in blue. Panels display (a) charging and (b) discharging behavior across the tested cation environments and mediator concentrations. Negative currents indicate discharge behavior, while positive currents correspond to charging behavior.

As clearly illustrated in Figure 4, the booster’s utilization in the lithium ferri/ferrocyanide and PB system is essentially negligible. Despite the theoretically available SoC window indicating that ideally 40% booster utilization is available, only about 1.5% of the booster’s capacity is actually utilized. This minuscule activity is further confirmed by current partitioning analysis, which reveals no distinct operational domains for the booster. The fluctuating booster current hovers close to zero, resulting in an overall utilization so low that the booster’s role in this system can be considered practically insignificant.

In contrast to lithium, the sodium system exhibits a markedly different behavior. During discharge, the current transferred to the booster remains consistently small relative to that associated with the mediator, across all tested concentrations. However, during charging, the booster significantly aids the oxidation of the mediator, whereas the desired mechanism in practical operation is mediator regeneration through booster reduction during the charge cycle. This effect is especially noticeable at a mediator concentration of 100 mM. This booster effect diminishes as the mediator concentration increases, yet, as emphasized earlier, the booster’s influence is decidedly stronger during discharge. Although initially modest, the booster current steadily increases throughout the discharge process, highlighting a progressive booster activation.

Most strikingly, the potassium system exhibits two distinctly defined operating regions, consistent with a pronounced booster role. Up to 50% SoC, the booster current is small and stable, but beyond this threshold, a substantial surge occurs, suggesting that a significant fraction of the applied current is associated with booster participation rather than mediator processes (as shown in Figure 2b). Furthermore, the charging rate profiles in Figure 2a suggest that up to 40% mediator SoC, the booster actively assists in further charging the mediator alongside the applied current. A parallel effect, seen in the sodium system at lower concentrations, also appears here: instead of facilitating the desired regeneration of the mediator, the booster seems to accelerate mediator oxidation. Importantly, this “undesirable” reaction manifests at all tested concentrations for the potassium environment, although its intensity diminishes as mediator concentration increases. Following this, the system displays a sharp peak in the redox-mediated reaction rate around 75% SoC, after which a sustained steady state is maintained until the end of the cycle. Taken together, these distinct behaviors are consistent with a strong and multifaceted involvement of the booster in the potassium system’s indirect electrochemical processes. Such clear demarcation and substantial booster participation make these findings fundamentally important for understanding and optimizing redox-mediated systems. Moreover, these insights can inform the optimization of operating conditions, providing a strong basis for practical RMFB adaptations.

### Booster Resistance and Ion Diffusion

It has been observed that the nature of the intercalating ion significantly influences the redox-mediated reactions between PB and ferri/ferrocyanide aqueous systems. The differences in system behavior may be explained by conductivity changes in PB during charging and discharging, as well as by variations in the ionic radii of the intercalating cations. Phase transition studies on MnHCF reveal that smaller ions occupy lower-energy positions at the center of available sites, causing lithium and sodium ions to displace toward these centers, which alters the crystal structure and reduces ionic conductivity.^63,64^ Although PB booster pellets were integrated with conductive carbon black to enhance ionic transport, it remains important to explore the possibility that conductivity changes within the pellets, dependent on the intercalating cation, may reduce overall booster utilization.

Aiming to compare the booster’s resistance and intercalation dynamics in three different cation environments, EIS is done on booster coated glassy carbon electrode presented in Figure 6. The high-frequency region is typically associated with the ohmic resistance, which includes contributions from the electrolyte, electrode, and wiring. The diameter of the semicircle represents the charge transfer resistance, reflecting how easily ions and electrons can move across the electrode interface. The low-frequency region is characterized by the Warburg impedance, which corresponds to the solid-state diffusion of ions within the electrode material. The length and slope of this Warburg tail provide insight into the ease or difficulty of ion diffusion through the lattice.^65^

**Figure 6:**
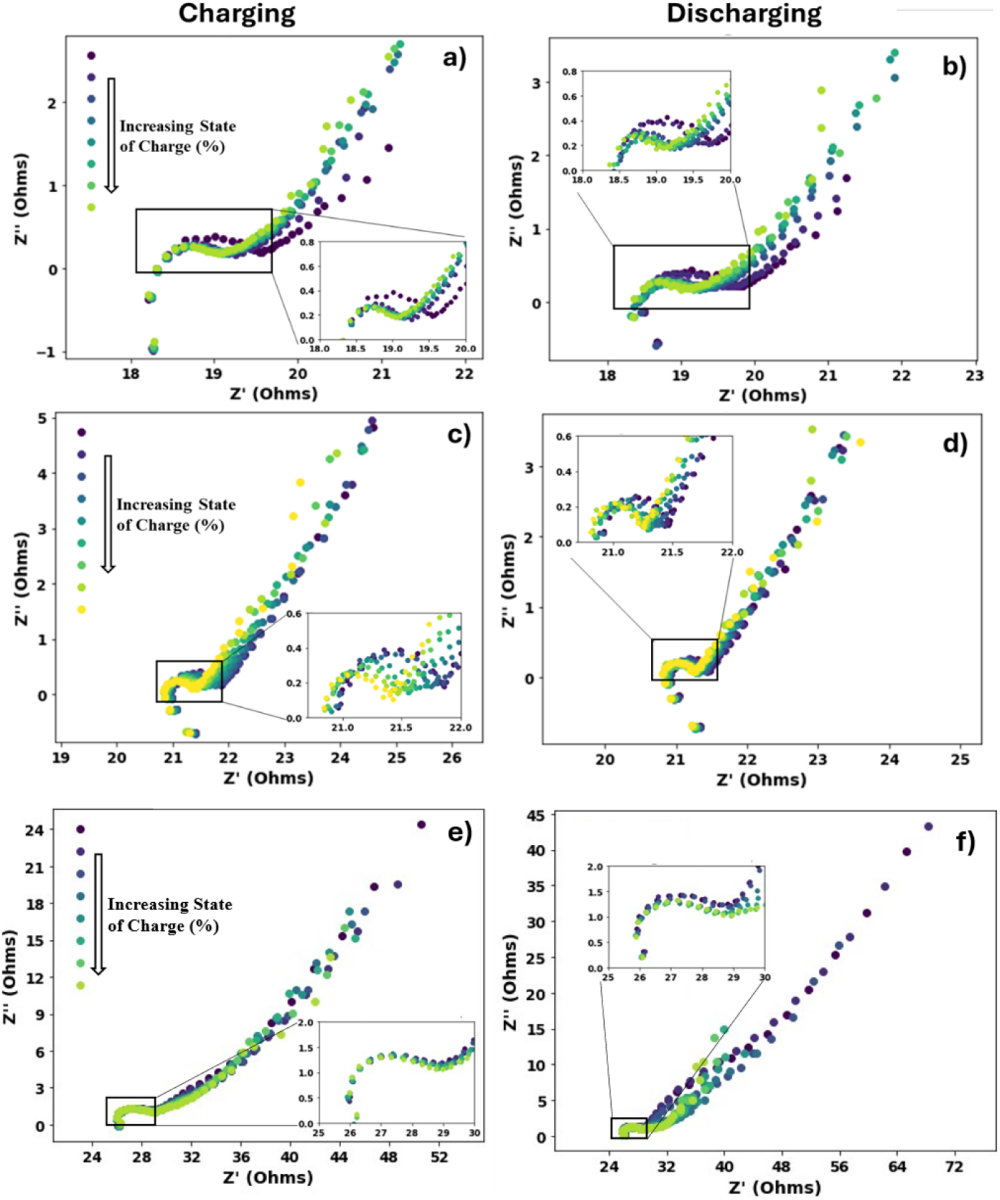
Electrochemical impedance spectroscopy (EIS) profiles of Prussian blue-coated glassy carbon electrode at varying SoC with a–b) K^+^, c–d) Na^+^, e–f) Li^+^ as the intercalating ion, showing changes in total resistance during charging and discharging.

The Nyquist plots in Figure 6 demonstrate systematic changes in impedance as the electrode progresses through increasing and decreasing states of charge. Although the semicircles in the EIS spectra shift slightly to the right with higher state of charge, the overall change in resistance remains minimal. This indicates that the state of charge has little influence on the resistance of booster pellets, likely due to the enhanced conductivity imparted by the integrated carbon black. Diffusion within the PB lattice is effectively reflected by the Warburg tails in the low-frequency region of the spectrum. Notably, a longer Warburg tail corresponds to a greater Warburg coefficient and thus slower ion diffusion. Examination of the EIS profiles reveals that the lithium system exhibits the longest Warburg tail, indicating the slowest diffusion. The sodium system displays a shorter tail than lithium, while the potassium system presents the shortest tail, signifying the most efficient ion diffusion among the three.

Additionally, the low utilization of PB in aqueous RMFB systems where Li^+^ and Na^+^ serve as the intercalating cations can be attributed to lattice distortions caused by the larger sizes of Na^+^ and Li^+^ which exhibit a reduced propensity to dehydrate prior to intercalation in contrast to K^+^. Regarding the cycling stability of PB in aqueous batteries, cations with low hydration energies such as K^+^ have proven to be the most effective candidates for enhanced cycling stability. This is because K^+^ can shed its hydration shell more readily than Li^+^ and Na^+^, which remain more strongly solvated, hindering efficient intercalation and deintercalation.^53^

However, the presence of two distinct regions in the redox-mediated reaction rate observed for the K^+^ system remains unexplained despite extensive literature review and electrochemical impedance spectroscopy (EIS) measurements.

### Distinct Redox Regions Linked to Booster SoC in Potassium RMFBs

Nyquist plots of the booster, together with literature-reported diffusion and intercalation characteristics of aqueous cations, elucidate the observed trends in booster utilization. Na^+^ and Li^+^ being larger cations upon intercalation that disrupt the PB lattice,^53^ Li^+^ to the greatest extent, also diffuse inside PB more slowly than K^+^. In contrast, the K^+^ system not only mediates more effectively but also appears to exhibit lower resistance values and benefits from its smaller size upon intercalation/deintercalation. However, these resistance and diffusion characteristics alone do not fully account for the redox mediation rate trends observed in Figure 5. To gain deeper insight, attention was turned to the SoC profiles of both the mediator and the booster as a function of potential to illuminate the origins of the distinct redox-mediated reaction rate regions observed in K^+^ intercalating RMFBs. Figure 7 highlights how specific regions in the SoC versus potential profiles (indicated by blue line and red dashed lines) correspond to pronounced features in the redox-mediated reaction rate curve (red-blue-green markers).

**Figure 7:**
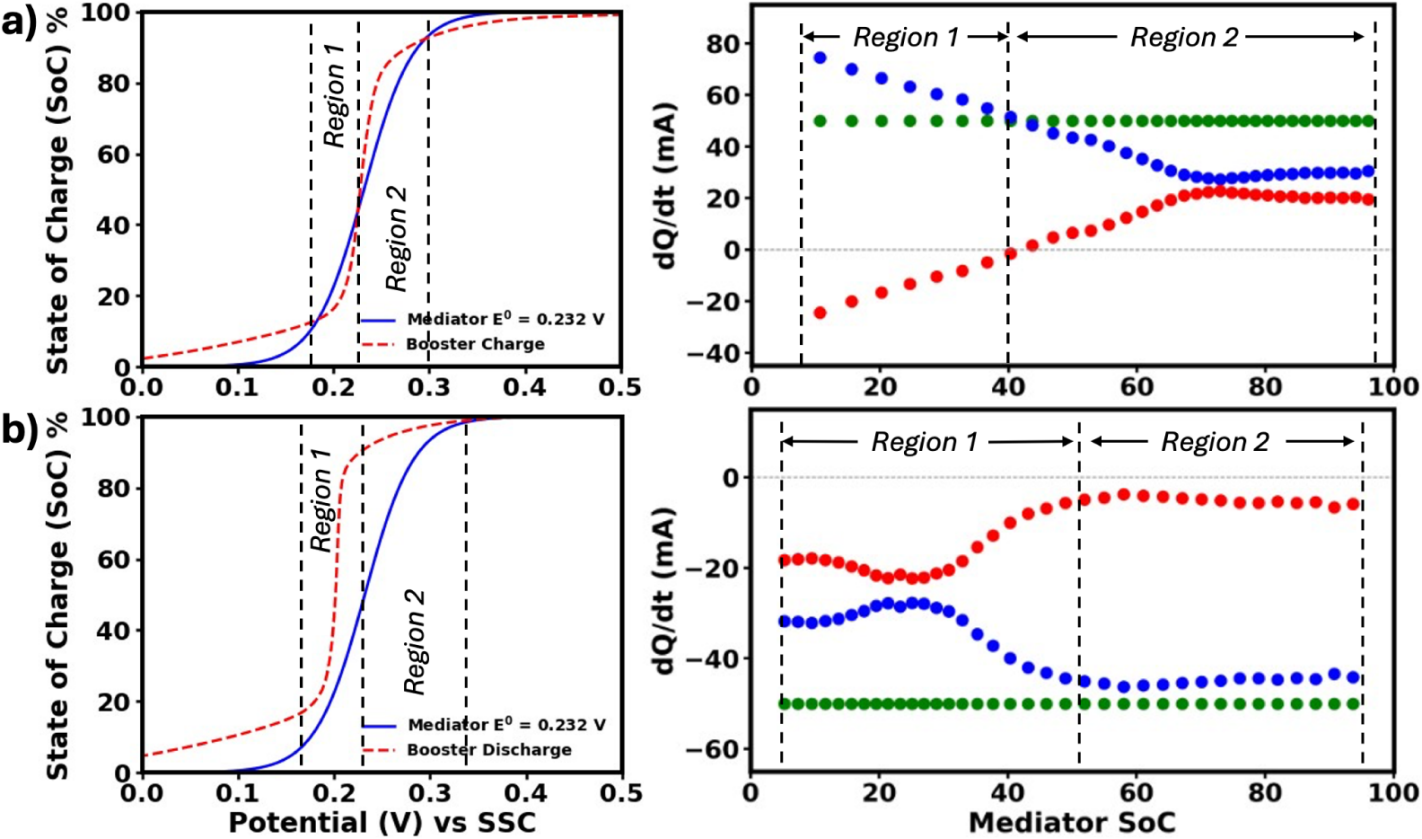
a) Charge, b) discharge curves of the booster and mediator in a K+ environment, shown alongside the corresponding redox mediation rate graphs. The d*Q*/d*t* vs mediator SoC plots illustrate the total current (green markers) and its division into current consumed by the mediator (blue markers) and current transferred to the booster (red markers). When boosters are introduced, they are fully oxidized. This potential difference causes a portion of the mediator to be oxidized immediately, resulting in a first charge capacity that is smaller than the subsequent discharge capacity. Therefore, d*Q*/d*t* graphs are derived from the data collected on the first discharge and the following charge cycle.

Notably, when the mediator’s SoC curve is modeled using the Nernst equation (i.e., under ideal thermodynamic assumptions), the initial rise (0%–5% SoC) in the mediator’s SoC curve (blue line) is so abrupt that these regions provide little opportunity for effective indirect electron transfer, thereby restricting booster utilization from the outset. This steep mediator activation further accentuates the limitation of the booster’s contribution in these crucial segments. In contrast, the SoC behavior of the booster (red dashed line) deviates noticeably from the ideal Nernstian response, generating non-uniform redox-mediated reaction rate regions, an observation that challenges conventional expectations for booster performance.^46^

During charging (Figure 7a), the “undesirable” effect of the booster observed in Region 1 can be rationalized by considering its state at the end of the previous discharge cycle. The booster was initially introduced in a fully charged state (100% SoC), but if it is only discharged to about 50% SoC through indirect mediation, its potential remains relatively high. As a result, at the start of the next charging step, the booster’s potential is higher than that of the mediator, creating an unfavorable imbalance that promotes mediator oxidation rather than efficient redox mediation.

Kinetic limitations further reinforce this scenario. As shown in Figure 7b, the current associated with the booster during discharge remains relatively small compared to that of the mediator (blue marker in Figure 7b), indicating restricted booster involvement due to both limited discharge depth and to the slower redox-mediation kinetics compared with the direct electrode reaction. Supporting data (exchange current density values, Figure S2, Table S1) suggest that the mediation reaction cannot fully keep pace with the electrode process, causing the booster’s potential to “lag” behind the mediator. Together, these factors help explain why the booster’s effect in Figure 7a—Region 1 deviates from ideal mediated behavior and highlight the dual importance of achieving full discharge and overcoming kinetic barriers to optimize booster utilization in potassium-ion intercalating RMFBs.

Current associated with the booster appears positive in Region 2 as shown in Figure 7a, indicating that the booster now can regenerate the mediator for further oxidation. Assuming the booster was not fully discharged in the previous cycle, comparison of the booster and mediator SoC curves shows that, in Region 2, the mediator reaches a potential higher than that of the booster, enabling the booster to be charged.

Moreover, a critical insight emerges during discharge: the SoC profiles of the booster and mediator reveal that, in Region 2 of Figure 7b, a change in mediator SoC corresponds to only about 10% change in booster SoC. In this region, the booster’s current contribution remains minimal compared to that of the mediator, simply because the accessible SoC window for the booster is so limited. By contrast, in Region 1, the booster exhibits a much higher current response. Here, the booster’s SoC curve, as noted earlier, has a considerably flatter profile, allowing its SoC to change by as much as 70% while the mediator SoC decreases from 50% to 5%. This significantly larger accessible capacity in Region 1 enables a higher rate of the indirect, redox-mediated reaction. Such findings further illuminate how the system’s electrochemical dynamics, governed by both thermodynamic and kinetic factors, determine the efficiency and activity of the booster under varying cycling conditions.

### Design Implications

Overall, the results indicate that Na^+^ and Li^+^ in aqueous redox flow batteries with a Prussian Blue booster and ferri/ferrocyanide mediator do not facilitate redox mediation as effectively as K^+^. Achieving higher energy densities in RMFBs therefore requires careful consideration of mediator concentration, electrolyte composition, and redox mediation kinetics. Literature reports show that lithium ferri/ferrocyanide exhibits higher solubility in aqueous media than its sodium or potassium analogs, enabling greater achievable capacities within solubility limits.^55^ Enhancing the capacity of an energy-dense mediator such as lithium ferri/ferrocyanide could maximize the benefits of redox targeting in flow batteries; however, a clear trade-off exists between mediator solubility (higher for Li^+^) and potential alignment (optimal for K^+^). Building on these insights, prospects for multication electrolytes or mixed-ion boosters appear particularly promising. Analysis of the SoC windows in which the booster is active across different cations suggests that mixed-electrolyte strategies could suppress unwanted mediation reactions during charging, thereby improving booster utilization and overall system performance.

## Conclusion

This study examined the impact of cation size on redox-mediated reactions in aqueous RMFBs to define thermodynamic limits, practical booster utilization, and redox mediation kinetics. Potassium-based systems showed the most favorable behavior, with booster utilization improving as mediator concentration increased, consistent with prior observations. In contrast, sodium and lithium systems exhibited limited capacity gains even at higher mediator concentrations.

Reaction rate analysis using operando UME CV and EIS further explained these trends. While booster resistance remained largely unchanged, diffusion within the PB lattice was significantly slower for sodium and lithium compared to potassium, accounting for their poorer performance. The superior kinetics and utilization window of potassium systems highlight its advantage over other cations.

Achieving higher energy densities in RMFBs will therefore depend on optimizing mediator concentration, electrolyte composition, and redox mediation kinetics, with potassium emerging as the most promising candidate among the studied systems.

## Materials and Methods

Sodium ferrocyanide decahydrate, pure Prussian blue, potassium ferrocyanide trihydrate, and lithium hydroxide were obtained from Thermo Scientific Chemicals. Sodium chloride, potassium chloride, lithium chloride, and hydrochloric acid (37%) were purchased from Sigma-Aldrich. 1-Methyl-2-pyrrolidinone was sourced from Fisher Chemical. Poly­(vinylidene fluoride) (PVDF) powder was acquired from Alfa Aesar, and TIMCAL Super C65 Conductive Carbon Black was provided by MSE Supplies. All chemicals were used as received without further purification.

### Electrolyte Synthesis

Lithium ferrocyanide was prepared as reported by Yu et al.^66^ Prussian Blue powder was reacted with 1 M LiOH in deionized water. After 10 h, a 0.2 M lithium ferrocyanide solution was obtained, with a brown precipitate forming at the bottom. The precipitate was removed by vacuum filtration using glass fiber filters.

A three-electrode setup was used for the oxidation of 0.2 M sodium ferrocyanide and lithium ferrocyanide, employing a carbon mesh working electrode, an Ag/AgCl reference electrode, and a platinum counter electrode. To oxidize 60 mL of 0.2 M sodium ferrocyanide, a constant current of 70 mA was applied with a 0.6 V potential cutoff. Following electrolysis, the solution pH was adjusted to neutral using concentrated hydrochloric acid.

### Electrolyte Preparation

For the flow cell experiments, electrolytes were prepared separately for each cation environment-potassium, sodium, and lithium-by diluting 0.2 M stock solutions in deionized water to obtain target concentrations of 0.1, 0.075, or 0.05 M for both ferricyanide and ferrocyanide with a total concentration of 0.2, 0.15, and 0.1 M active species, along with 1 M of the corresponding chloride salt.

### Solid Booster Preparation

The slurry was formulated by combining Prussian blue powder, carbon black, and PVDF in a mass ratio of 70:20:10, respectively. *N*-Methyl-2-pyrrolidone (NMP) was then added at approximately 4 g per gram of solid mixture. This blend was manually stirred with a spatula until a uniform consistency was achieved. The resulting slurry was dispensed into silicone molds, each forming half-spheres with dimensions of 0.5 in. in diameter and 0.5 in. in depth. The molds were heated overnight on a hot plate at 100 °C to facilitate NMP evaporation.

**Figure 8:**
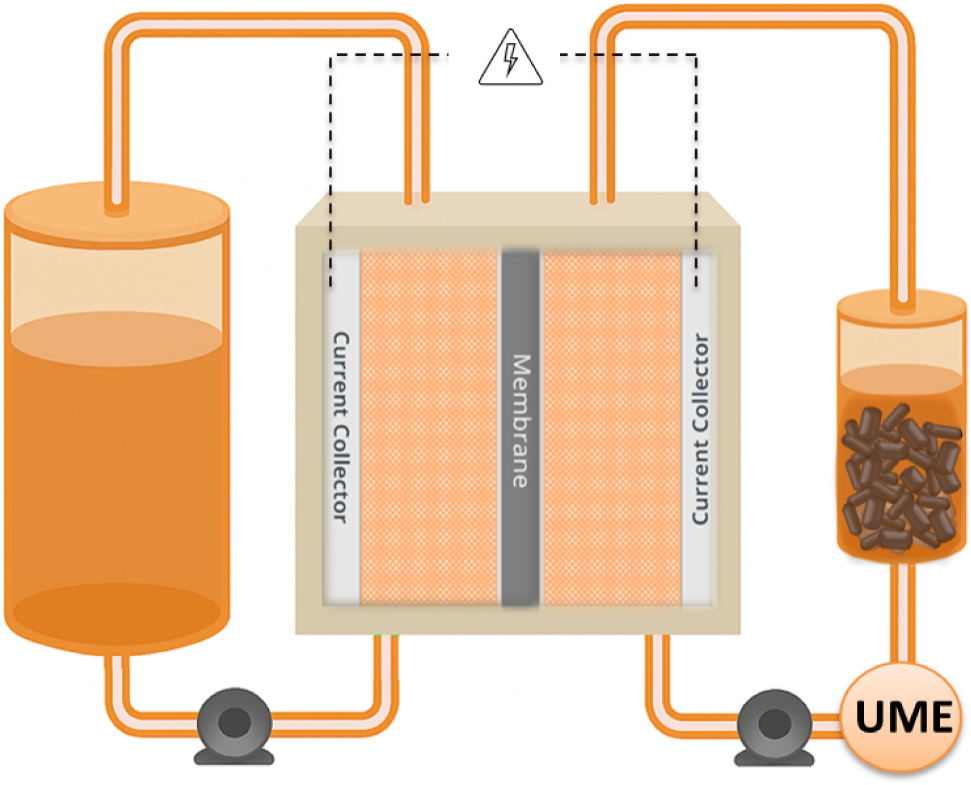
Schematic of the compositionally symmetric volumetrically asymmetric RMFB used in this study.

### Electrochemical Setups

Galvanostatic cycling experiments were performed using a flow cell design that maintained compositional symmetry but utilized differing electrolyte volumes on each side (Figure 8). Both reservoirs started at a 50% SoC with 25 mL of electrolyte on the capacity-limiting side and 95 mL electrolyte on the noncapacity-limiting side. Solid booster materials were added exclusively to the capacity-limiting reservoir after establishing baseline capacity (Figure S1).

All experiments utilized a 5 cm^2^ Scribner flow cell fitted with interdigitated flow fields, 3.4 mm thick AvCarb G300A carbon felt electrodes, and a Nafion 212 membrane. Electrodes were compressed to approximately 70% of their original thickness using two gaskets per side-one 1/16″ thick and one 1/32″ thick. The Nafion 212 membrane was preconditioned by soaking in 1 M KCl solution at 80 °C for 40 min, followed by immersion in deionized water at 80 °C for 24 h. Electrolytes were circulated at a flow rate of 20 mL/min using MasterFlex peristaltic pumps. Charge and discharge cycles were carried out on a BioLogic VSP potentiostat at a constant current density of 10 mA/cm^2^, with a voltage cutoff set at ±0.5 V.

### Cyclic Voltammetry

To obtain mediator profiles, 10 mL of electrolyte containing 0.1 M active material and 1 M supporting salt was used. Carbon rods served as both the working and counter electrodes, while a silver/silver chloride (SSC) electrode in 1 M KCl acted as the reference. Cyclic voltammetry (CV) was performed at a scan rate of 5 mV/s over a potential window of −0.2 to 0.6 V vs SSC.

For experiments with solid boosters, the working electrode was a glassy carbon electrode (3 mm diameter) coated with the booster slurry, maintaining the same composition as the booster pellets. After coating, the electrode dried overnight at 80 °C and then immersed in 10 mL of 1 M supporting salt solution. The reference and counter electrodes were the same as in the mediator experiments. CV profiles were recorded at 5 mV/s over a potential window of −0.2 to 0.6 V vs SSC as in obtaining the mediator profiles.

### Charge/Discharge Profiles

The charge/discharge curve for potasium ferri/ferrocyanide is obtained via plugging in the formal potential (*E* ^0^), obtained from CV, to the Nernst equation where *R* is the gas constant, *T* is temperature and *n* as the number of electrons transferred.

4 $${E=E}^{0}+\frac{RT}{nF}\ln\left(\frac{\mathrm{SoC}}{1-\mathrm{SoC}}\right)$$

SoC curves for booster in Figures 2 and 3 are obtained by using a similar booster coated glassy carbon electrode and three electrode system with carbon rod counter, SSC reference electrode. 1 M KCl, NaCl, LiCl were the solutions used for constant current charge and discharge by applying 0.05 mA with a voltage cutoff set at 0.6 V to −0.2 V for K^+^ and Na^+^ environment, 0.6 V to −0.4 V were cutoff voltages in Li^+^ environment.

### Electrochemical Impedance Spectroscopy

The setup, similar to that used for cyclic voltammetry, was employed. Prior to obtaining Nyquist plots, the working electrode was conditioned at constant potentials corresponding to various SoC of the booster (Figure S4, Figure S5, Figure S6) for 10 min. Following each potential conditioning, potentiostatic electrochemical impedance spectroscopy (PEIS) is performed. EIS measurements were performed using a Biologic potentiostat/galvanostat across a frequency range of 160 to 1 Hz with an amplitude of 5 mV at open circuit potential.

### Operando Ultramicroelectrode Cyclic Voltammetry

A carbon disk ultramicroelectrode (UME, 11 μm diameter) was installed in-line on the capacity-limiting side of the flow cell, accompanied by an SSC reference electrode and a glassy carbon rod serving as the counter electrode. CV measurements were recorded continuously throughout the cell’s charging and discharging process. The voltage window for these scans was set from 0 to 600 mV vs SSC using a scan rate of 5 mV/s.

### Theoretical Booster Capacity and Current Partition Calculations

The theoretical specific capacity of Prussian Blue, as reported in previous studies from both our group and the literature, is 124 mAh/g and is used here to calculate booster utilization.^38,61^

Quantification of mediator concentration from the steady state voltammograms and current for booster and mediator upon flow battery conditions are performed as reported by Rourke et al.^61^

## Supplementary Material



## Notes

Flow battery cycling data demonstrating capacity increase; open-circuit potential (OCP) analysis with exchange current density evaluation between Prussian blue and potassium ferri/ferrocyanide; potential conditioning procedures; electrochemical impedance spectroscopy (EIS) sampling points (PDF)
