# Supplementary material for: Influence of Alkali Cations on Redox Matching and Capacity Access in Redox-Mediated Flow Batteries
Source: ACS Omega. 2025 Dec 31;11(1):1335–47. doi: 10.1021/acsomega.5c08835 (PMC12809325; doi:10.1021/acsomega.5c08835)
Supplement: Supplementary file 1 [file ao5c08835_si_001.pdf]

Influence of Alkali Cations on Redox Matching and Capacity Access in Redox-Mediated Flow Batteries

Eylul Ergun[1], Daniel Rourke[1], Shabdiki Chaurasia[1], Tulsi Poudel[2], Patrick Cappillino[2], Ertan Agar[1*]

[1]Department of Mechanical Engineering, Energy Engineering Graduate Program, University of Massachusetts Lowell, Lowell, MA 01854 USA
[2]Department of Chemistry and Biochemistry, University of Massachusetts Dartmouth, Dartmouth, MA 02747 USA
[*] Corresponding author: Ertan_Agar@uml.edu

Flow Battery Cycling and Capacity Increase

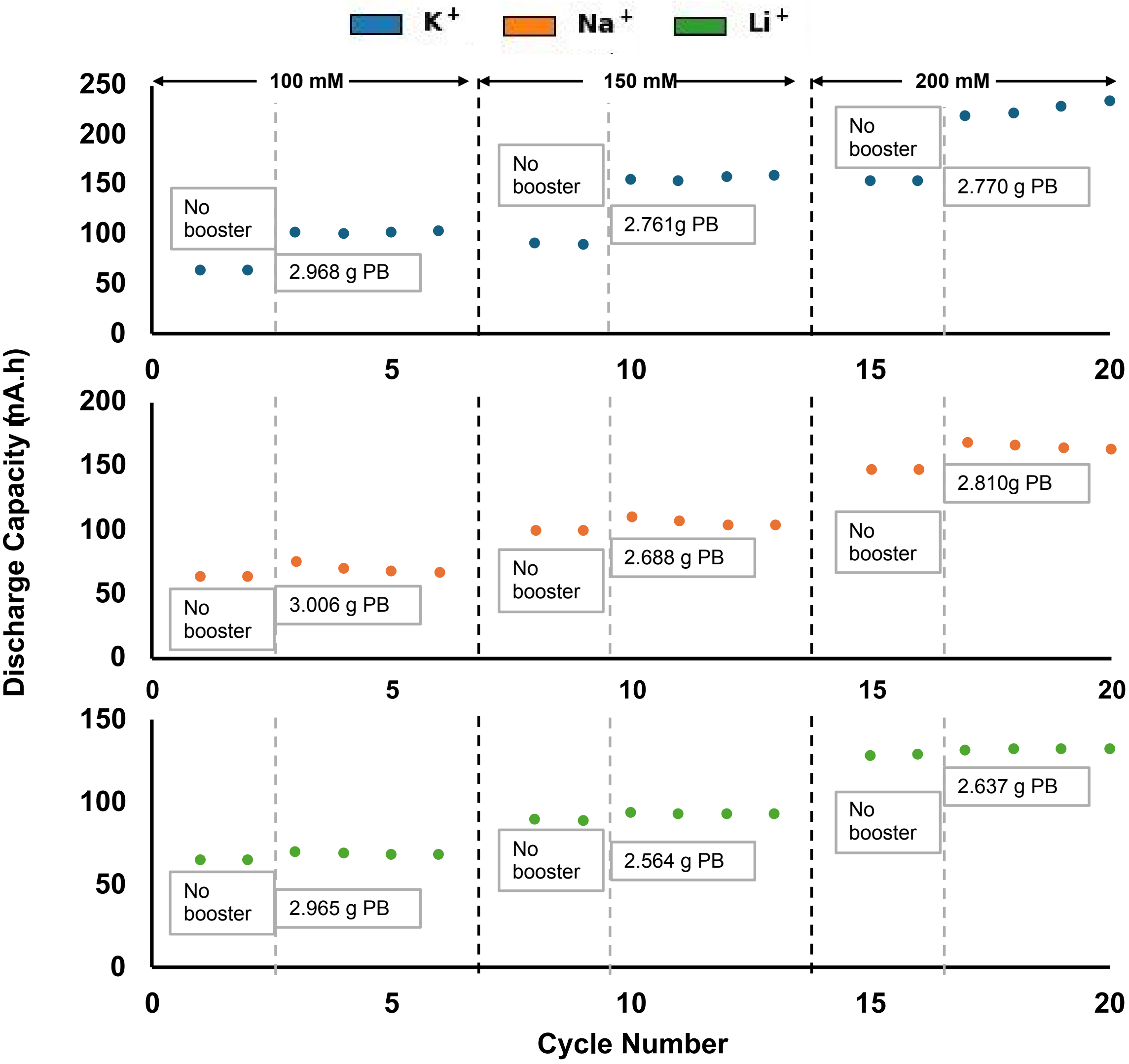

Figure S1 Charge/discharge capacity for mediator analogues and the effect of increasing mediator concentration Cycles without associated data points are shown as is, because during those periods deionized water was circulated before introducing a fresh electrolyte containing a different mediator concentration into the external tanks. Each black dashed line, therefore, marks the conclusion of one experiment in the concentration study.

The flow battery is cycled twice prior to booster addition to establish a baseline mediator capacity. On the third cycle of each experiment (i.e., cycles 3, 10, and 17 in **Figure S1**), 2.5–3.0 g of booster pellets are introduced. For consistent comparison across experiments, the discharge capacity from the fourth cycle after booster introduction (cycles 6, 13, and 20 in **Figure S1**) is used for analysis. Booster utilization and capacity enhancement are calculated as the difference between the discharge capacity of this fourth cycle (with booster) and the second cycle (baseline, without booster).

Open-Circuit Potential Analysis and Kinetics

The redox mediation reaction was found to persist even after the scheduled charge–discharge cycles had concluded, indicating ongoing mediator activity in the absence of an applied current. This observation prompted open-circuit potential (OCP) experiments and in situ exchange current density calculations in redox-mediated flow batteries under no applied current conditions, following methodologies similar to those previously reported.[1,2]

The current arising from the redox mediation reaction between the ferrocyanide mediator and the Prussian blue booster is defined as the effective current (i_{eff}) in Equation 2. It is calculated as the rate of change of charge with respect to time ($\frac{dQ}{dt}$), where the charge (Q) is tracked by measuring the concentration change of the oxidized mediator species over time using ultramicroelectrode cyclic voltammetry (UME CV). This concentration change per unit time is then converted to charge by multiplying by Faraday's constant (F) and the volume of the electrolyte (V).

$$i_{eff} = \frac{dQ}{dt} = FV\frac{dC_{ox}}{dt}(1)$$

Overpotential (η) for the booster is then defined by the relation in Equation 2. From the cyclic voltammetry experiments we were able to establish $E_{1/2}^{booster}$. In our case, the open-circuit potential (OCP) is readily obtained by substituting the mediator concentration into the Nernst equation.

$$\eta = E_{1/2}^{booster} - (2)$$

Based on Butler-Volmer (B-V) electrode kinetics, the B-V model can be linearized for very small overpotentials.[3] Expressed as Equation 3, where R is the gas constant, T is the temperature, and f=F/RT, low-overpotential regime gives the relation between current and overpotential.

$$i_{eff} = -\, i_0(3)$$

The small overpotential conditions required for the linearized Butler-Volmer model are not met when using sodium and lithium ferri/ferrocyanide, as both exhibit large positive overpotentials. Instead, data from potassium-based environments were used. The exchange current density was normalized by the booster surface area to obtain values for the tests conducted at the lowest (100 mM) and highest (200 mM) mediator concentrations.

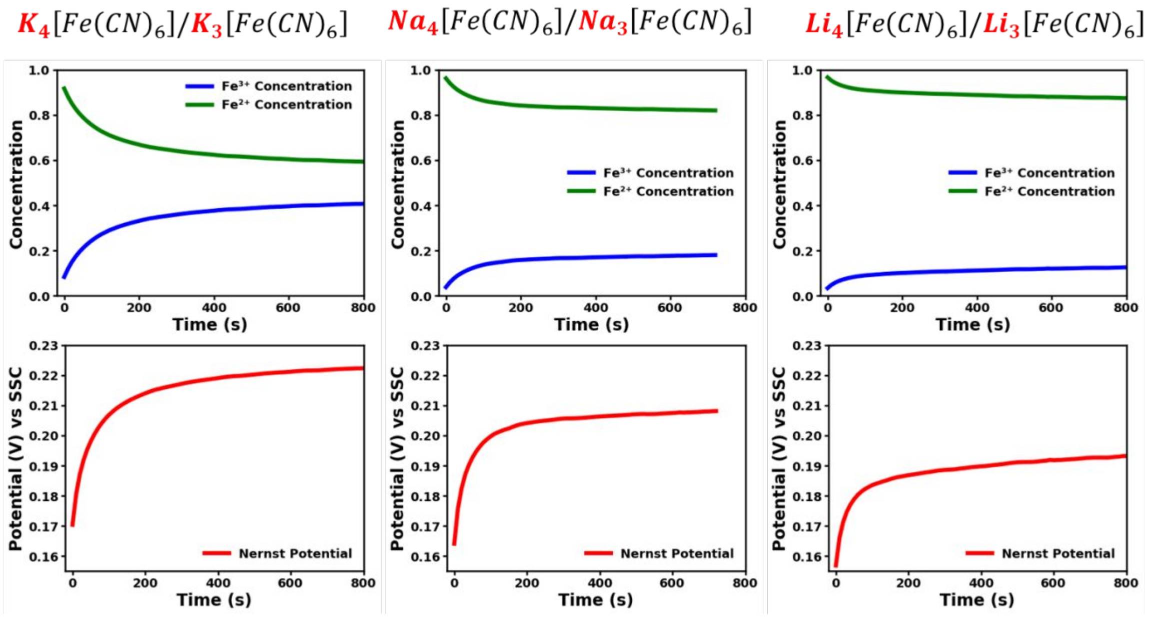

Figure S2 Change in concentration of the active material for 100 mM ferri/ferrocyanide analogues and the corresponding Nernst potential. The Nernst potential is calculated using the ratio of the oxidized (ferricyanide) to reduced (ferrocyanide) species concentrations.

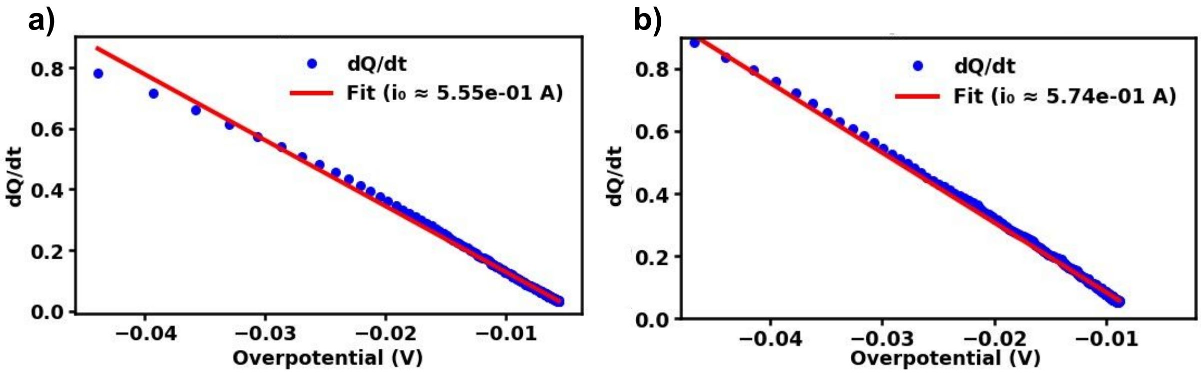

Figure S3 Simplified Butler-Volmer fitting for exchange current calculation for a)100 mM Potassium ferrocyanide and b)200 mM Potassium ferrocyanide.

**Surface area of PB**	**Mediator Concentration**	**Booster added**	**Exchange current**	**Exchange current density**

**60 m²/g**	**100 mM**	**2.986 g**	**0.55 A**	**3.07×10^{-4}mA/cm²**
**60 m²/g**	**200 mM**	**2.703 g**	**0.57 A**	**3.54×10^{-4}mA/cm²**

Table S1 Exchange current density of potassium ferri/ferrocyanide and prussian blue booster pellets and parameters used for calculation. Surface are of the booster is obtained by Brunauer-Emmett-Teller (BET) analysis.

To quantify the inherent redox mediation rate between ferri/ferrocyanide and PB in our system, we used exchange current density (j_0). The calculated j_0 values suggest that the current required for the reaction in the flow cell is on the order of 10^{-4} mA/cm^2. Given this, applying a current density of 10 mA/cm^2 at the electrodes results in an electrochemical reaction at the electrodes that is significantly faster than the redox mediation reaction occurring in the tank. This difference in rates highlights that the overall system performance is limited by the slower kinetics of the redox mediation process.

Potential Conditioning and Electrochemical Impedance Spectroscopy Sampling

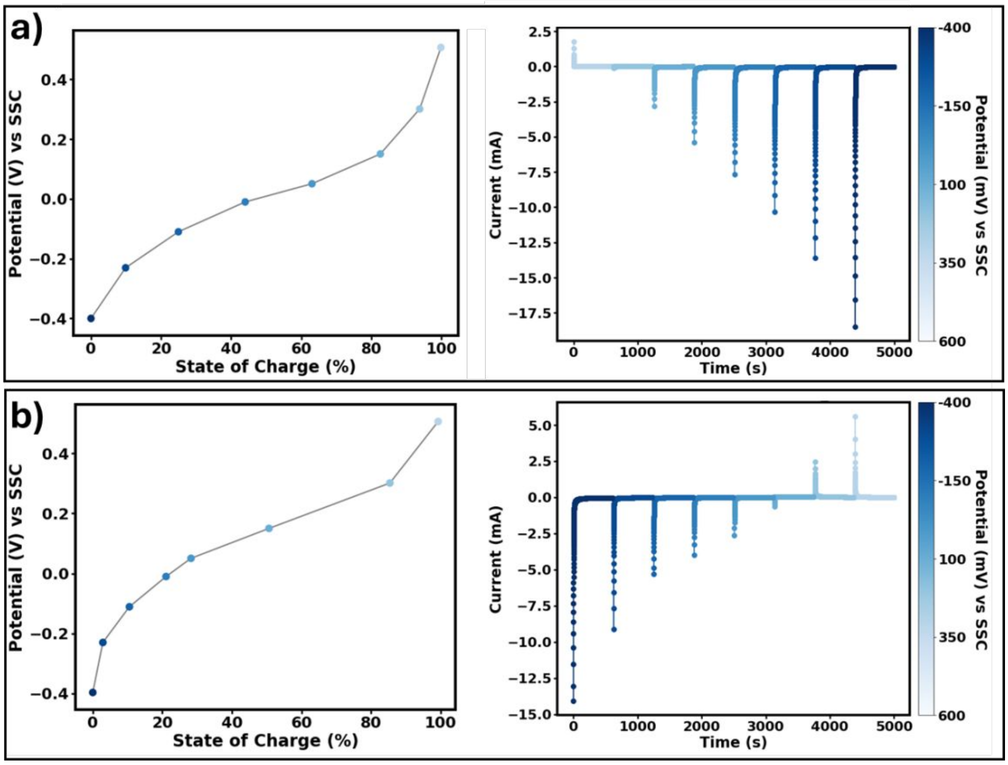

Figure S4 Sampling points on the a) discharge b) charge curve for EIS and corresponding current decay performing chronoamperometry at the sample potentials for booster in 1M LiCl.

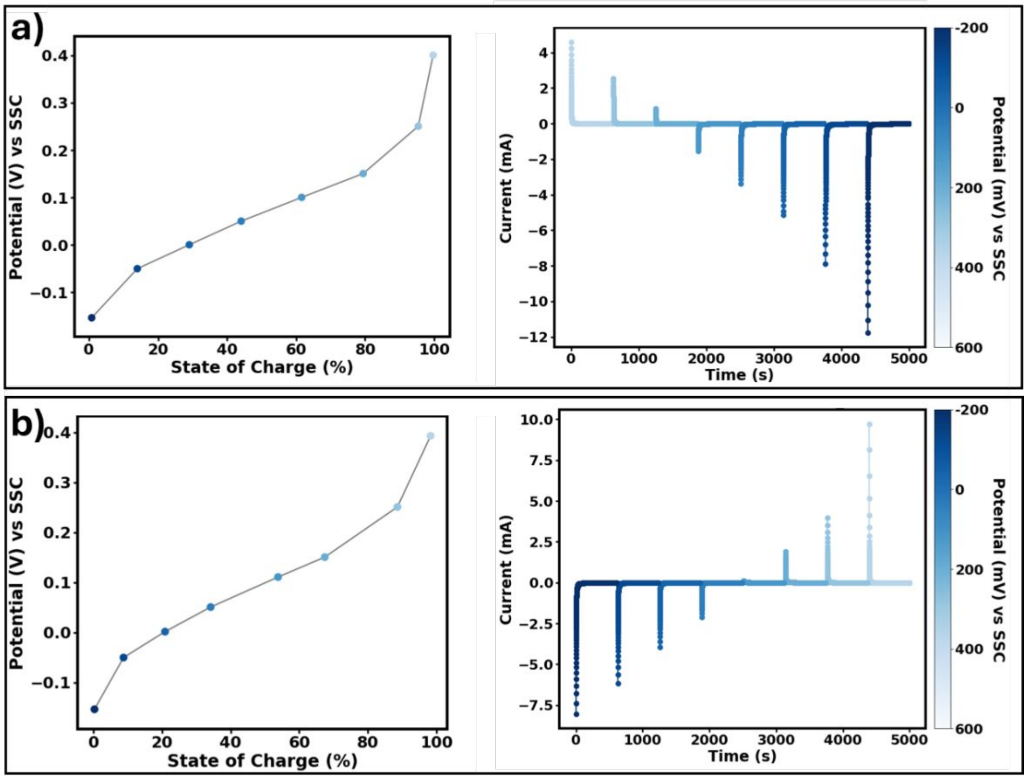

Figure S5 Sampling points on the a) discharge b) charge curve for EIS and corresponding current decay performing chronoamperometry at the sample potentials for booster in 1M NaCl.

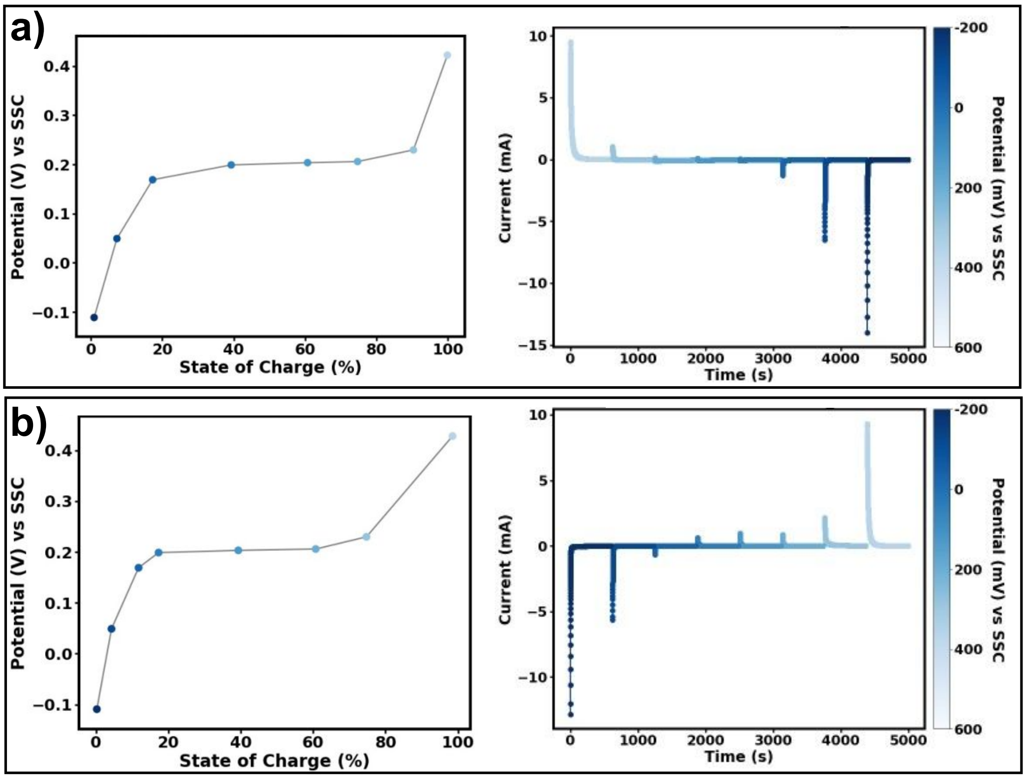

Figure S6 Sampling points on the a) discharge b) charge curve for EIS and corresponding current decay performing chronoamperometry at the sample potentials for booster in 1M KCl.

References

(1) Huang, S.; Yuan, Z.; Salla, M.; Wang, X.; Zhang, H.; Huang, S.; Lek, D. G.; Li, X.; Wang, Q. A Redox-Mediated Zinc Electrode for Ultra-Robust Deep-Cycle Redox Flow Batteries. *Energy Environ. Sci.* **2023**, *16* (2), 438–445. https://doi.org/10.1039/D2EE02402K.

(2) Zhou, M.; Chen, Y.; Zhang, Q.; Xi, S.; Yu, J.; Du, Y.; Hu, Y.; Wang, Q. $Na_3 V_2 (PO_4)_3$ as the Sole Solid Energy Storage Material for Redox Flow Sodium-Ion Battery. *Adv. Energy Mater.* **2019**, *9* (30), 1901188. https://doi.org/10.1002/aenm.201901188.

(3) Bard, A. J.; Faulkner, L. R. *Electrochemical Methods: Fundamentals and Applications*, 2. edition.; Wiley: New York Weinheim, 2001.
